
JOURNAL INFORMATION
==============================
NLM Title Abbreviation: Curr Med Sci
Journal ID: Curr Med Sci
NLM Journal ID: 101729993

Current Medical Science

ISSN: 2096-5230
EISSN: 2523-899X

ARTICLE INFORMATION
==============================
PMCID: PMC8184548
PMID: 33582925
DOI: 10.1007/s11596-020-2280-3
Article ID: 2280
Article version: 1
Subjects: Erratum

Erratum to: CAR T Cell Therapy for Hematological Malignancies

Yang Xin
Wang Gao-xiang
Zhou Jian-feng
grid.33199.31 0000 0004 0368 7223 Department of Hematology, Tongji Hospital, Tongji Medical College, Huazhong University of Science and Technology, Wuhan, 430030 China
Electronic publication date: 2021 Feb 13
Print publication date: 2021
Volume: 41
Issue: 1
First page: 187
Last page: 187
Copyright: © The Author(s) 2021
License: Open Access This article is licensed under a Creative Commons Attribution 4.0 International License, which permits use, sharing, adaptation, distribution and reproduction in any medium or format, as long as you give appropriate credit to the original author(s) and the source, provide a link to the Creative Commons licence, and indicate if changes were made.
The images or other third party material in this article are included in the article’s Creative Commons licence, unless indicated otherwise in a credit line to the material. If material is not included in the article’s Creative Commons licence and your intended use is not permitted by statutory regulation or exceeds the permitted use, you will need to obtain permission directly from the copyright holder.
To view a copy of this licence, visit http://creativecommons.org/licenses/by/4.0/.
License URL: https://creativecommons.org/licenses/by/4.0/

Erratum for: CAR T Cell Therapy for Hematological Malignancies. 39 6 16 12 2019 874 882 Curr Med Sci 10.1007/s11596-019-2118-z 31845217
issue-copyright-statement: © Huazhong University of Science and Technology 2021

==============================
The original article can be found online at 10.1007/s11596-019-2118-z

